# Incidental Thoracic Aortic Kinking Mimicking Aortic Coarctation: A Case Report

**DOI:** 10.7759/cureus.112647

**Published:** 2026-07-14

**Authors:** Nada Boutbagha, Hanane Ikrou, Hind Serhane

**Affiliations:** 1 Department of Pulmonology, Centre Hospitalier Universitaire Mohammed VI d'Agadir, Agadir, MAR

**Keywords:** aortic coarctation, ct angiography, incidental finding, pseudocoarctation of aorta, thoracic aortic kinking

## Abstract

Thoracic aortic kinking, also known as pseudocoarctation of the aorta, is a rare congenital anomaly characterized by elongation and angulation of the aortic arch without significant luminal narrowing. We report the case of a 67-year-old man admitted for hematemesis and melena. CT pulmonary angiography performed during the diagnostic workup revealed a proximal right pulmonary embolism and an incidental marked angulation of the descending thoracic aorta at the level of the aortic isthmus, without evidence of stenosis or collateral circulation. In the absence of clinical and radiological features of true coarctation, the diagnosis of thoracic aortic kinking was established. Conservative management with clinical and radiological follow-up was adopted. This report highlights the importance of recognizing thoracic aortic kinking as a rare benign anomaly that may mimic true aortic coarctation and emphasizes the role of imaging in establishing the correct diagnosis and avoiding unnecessary intervention.

## Introduction

Aortic kinking, also referred to as pseudocoarctation of the aorta, is a rare congenital anomaly characterized by elongation, redundancy, and buckling of the aortic arch without significant luminal narrowing or hemodynamic obstruction [1,2]. Unlike true coarctation, pseudocoarctation typically does not produce a pressure gradient across the affected segment and is therefore generally considered a benign condition [2,3]. The anomaly is believed to result from incomplete embryologic compression and shortening of the dorsal aortic roots, leading to elongation and tortuosity of the aortic arch [3].

Most patients remain asymptomatic, and the condition is frequently discovered incidentally during imaging studies performed for unrelated indications [1,2,4]. Advances in non-invasive imaging modalities, particularly CT angiography and magnetic resonance angiography, have improved the recognition and characterization of this uncommon vascular abnormality [5,6]. Typical imaging findings include elongation and kinking of the aortic arch, usually at the level of the isthmus, in the absence of significant stenosis or extensive collateral circulation, which helps distinguish pseudocoarctation from true coarctation [2,3].

Although generally considered a benign anomaly, pseudocoarctation may occasionally be associated with serious complications, including aneurysm formation, aortic dissection, and compression of adjacent mediastinal structures [5,7]. Furthermore, several reports have described concomitant congenital cardiovascular abnormalities, particularly bicuspid aortic valve, emphasizing the importance of comprehensive cardiovascular evaluation in affected patients [3,4]. Owing to the potential risk of aortic complications, periodic clinical and imaging follow-up may be warranted in selected cases [5,6,8].

Given its rarity and the potential for diagnostic confusion with true aortic coarctation and other aortic arch abnormalities, awareness of pseudocoarctation remains essential for accurate diagnosis and appropriate management [2,3]. We report a case of aortic kinking diagnosed in an adult patient and discuss its clinical presentation, imaging findings, differential diagnosis, and management considerations. The case is noteworthy because thoracic aortic kinking was incidentally identified during the diagnostic workup for acute pulmonary embolism, initially raising concern for aortic coarctation. This uncommon presentation highlights the importance of recognizing this rare anatomical variant to avoid misdiagnosis and unnecessary interventions.

## Case presentation

A 67-year-old man, a former smoker (30 pack-years) with a remote history of treated pulmonary tuberculosis, presented to the emergency department with moderate hematemesis and melena that had progressively developed over two days, associated with asthenia and deterioration of his general condition. He denied chest pain, dyspnea, and syncope. On physical examination, blood pressure measurements were symmetrical in all four limbs, and oxygen saturation was normal on room air. Abdominal examination revealed epigastric tenderness and painful splenomegaly, while digital rectal examination confirmed melena. No cardiac murmur, signs of heart failure, or pulse deficits were detected, and peripheral pulses were symmetrical and palpable.

**Figure 1:**
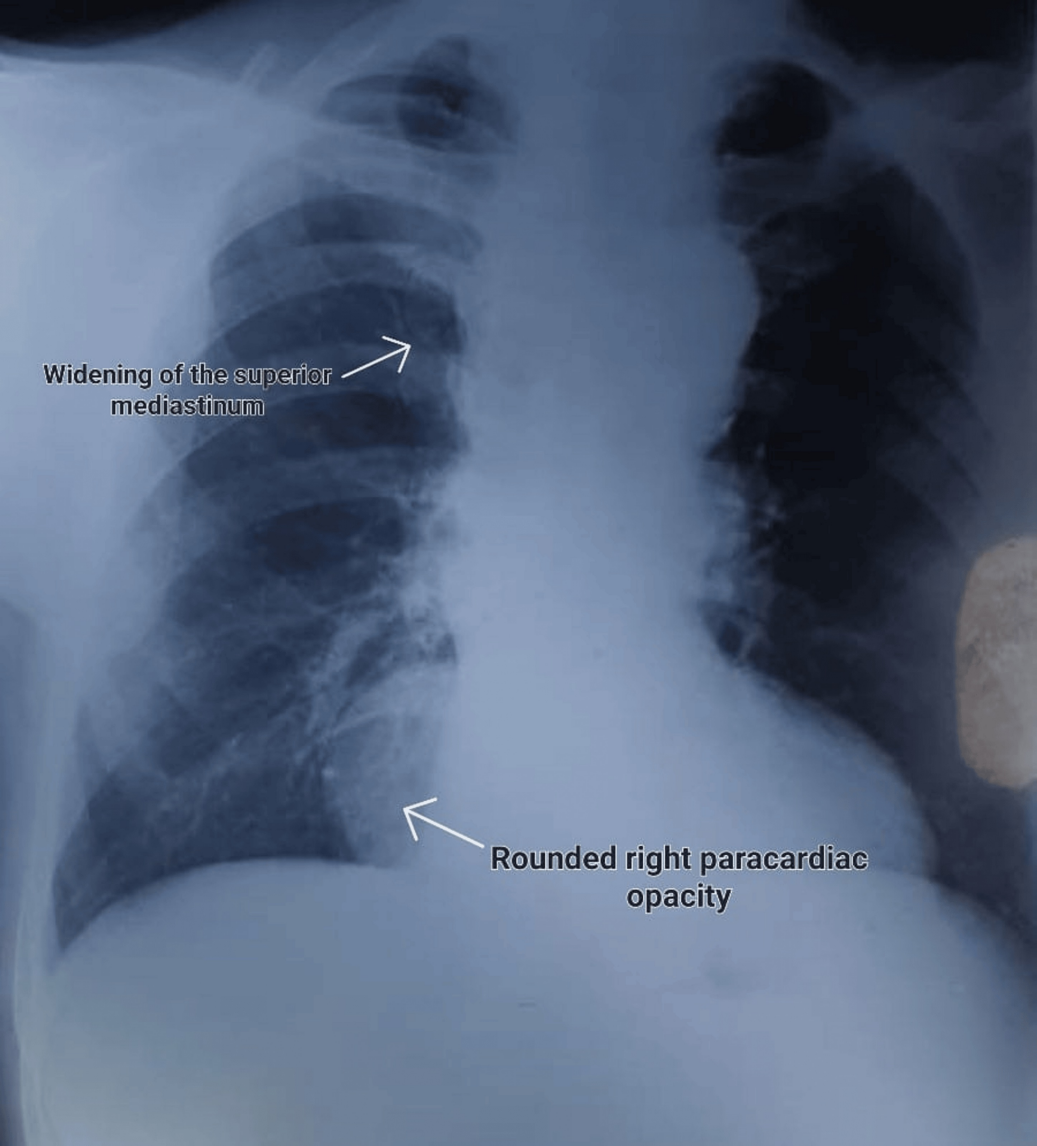
Chest radiograph findings The image demonstrates widening of the superior mediastinum (arrow) with a rounded right paracardiac opacity, findings that prompted further evaluation with contrast-enhanced CT to investigate a possible mediastinal vascular abnormality CT: computed tomography

The patient's vital signs and laboratory findings are summarized in Table 1. Laboratory investigations revealed anemia and elevated D-dimer levels, while inflammatory markers were only mildly increased and renal function was preserved. Electrocardiography showed normal sinus rhythm without significant abnormalities. Transthoracic echocardiography revealed mild dilatation of the proximal aorta and trivial aortic regurgitation. Left ventricular systolic function was preserved, with an estimated ejection fraction of 77%. No pericardial effusion or other significant structural cardiac abnormalities were observed. Chest radiography demonstrated widening of the upper mediastinum associated with a rounded right paracardiac opacity (Figure 1).

**Table 1:** Vital signs and laboratory findings at presentation Values were reported at the time of admission to the emergency department. BP: blood pressure; SpO₂: peripheral oxygen saturation; CRP: C-reactive protein

Parameter	Value
BP	125/75 mmHg
SpO_2_	99% on room air
Hemoglobin	8 g/dL
White blood cell count	6,250/mm³
Platelet count	304,000/mm³
CRP	9 mg/L
D-dimer	2,500 ng/mL
Renal function	Preserved

**Figure 2:**
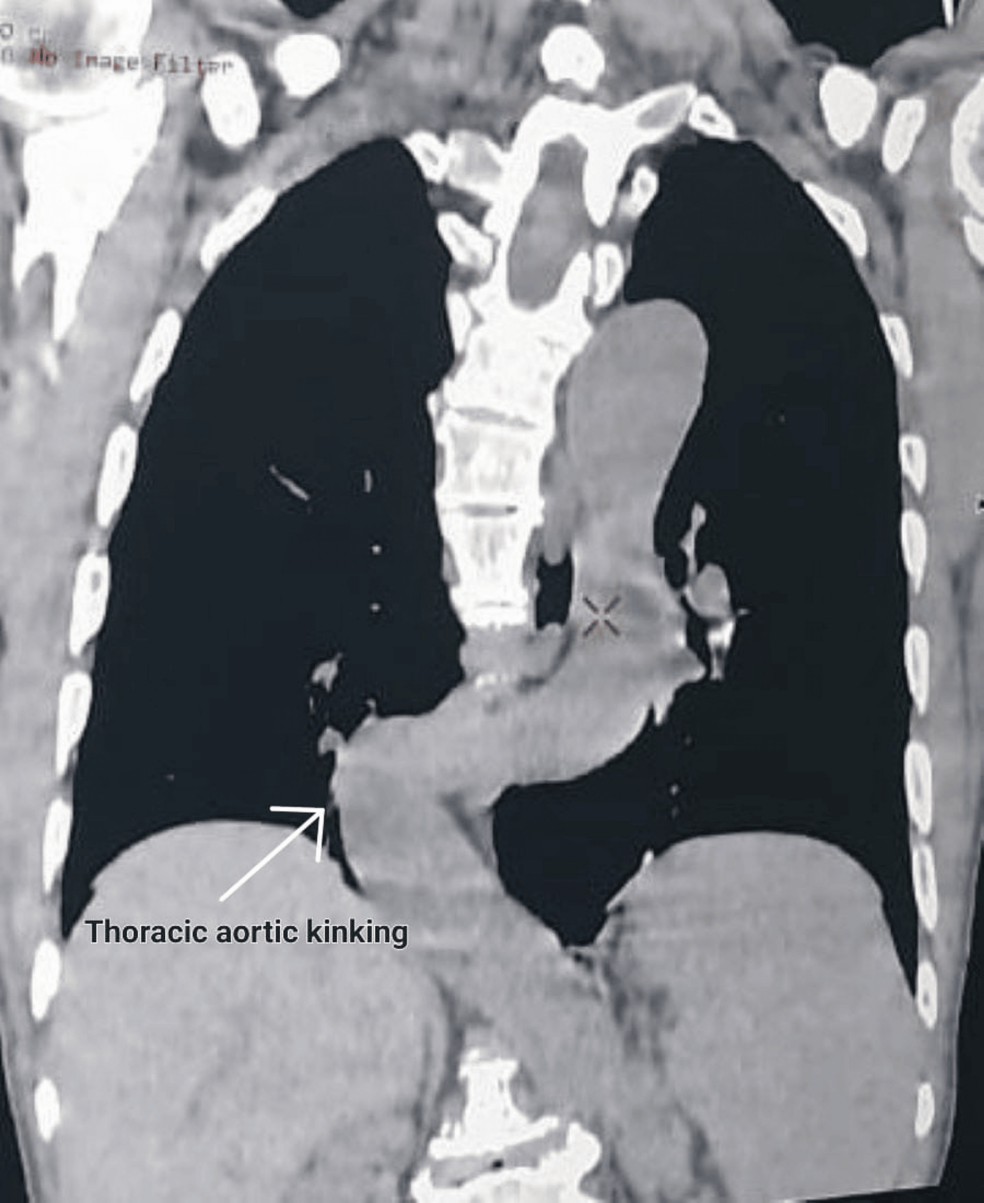
Coronal CT angiography findings The image demonstrates marked angulation (arrow) of the descending thoracic aorta at the level of the aortic isthmus, with preservation of the aortic lumen and no evidence of significant luminal narrowing or collateral circulation. These imaging findings are consistent with thoracic aortic kinking (pseudocoarctation) and help distinguish it from true aortic coarctation CT: computed tomography

Emergency esophagogastroduodenoscopy revealed neither active bleeding nor an identifiable upper gastrointestinal source of hemorrhage. Because of the elevated D-dimer level, CT pulmonary angiography was performed to investigate suspected pulmonary embolism. CT pulmonary angiography demonstrated a proximal right pulmonary embolism and incidentally revealed marked angulation of the descending thoracic aorta at the level of the aortic isthmus. No significant luminal narrowing or collateral circulation was observed. The absence of clinical signs of aortic obstruction, together with the lack of collateral vessel formation and the absence of a pressure gradient between the upper and lower extremities, argued against true coarctation and supported the diagnosis of thoracic aortic kinking (pseudocoarctation) [3,5]. The patient remained asymptomatic from a cardiovascular standpoint throughout hospitalization, and the aortic anomaly was considered an incidental finding unrelated to the patient's presenting symptoms (Figure 2).

Given the absence of hemodynamic compromise, arterial hypertension, aneurysmal dilatation, or other aortic complications, no specific intervention was indicated. Conservative management with periodic clinical and radiological follow-up was therefore recommended in accordance with current literature and existing guideline recommendations [3,5,6].

## Discussion

Aortic kinking, also referred to as pseudocoarctation of the aorta, is a rare congenital vascular anomaly most commonly attributed to abnormal embryological development of the aortic arch, resulting in elongation and redundancy of the vessel without significant luminal narrowing [1-3]. This condition is distinct from true coarctation of the aorta, as it is not typically associated with significant hemodynamic obstruction, systemic hypertension, or the development of collateral circulation [5,6].

From an imaging perspective, CT angiography and MRI represent the diagnostic modalities of choice, allowing precise anatomical characterization of the aortic arch. Typical findings include elongation and kinking at the level of the isthmus, preserved luminal diameter, and the absence of collateral vessel formation, which are key features in differentiating pseudocoarctation from true coarctation [3,4,8]. In our patient, these imaging criteria were clearly present, supporting the diagnosis and excluding a hemodynamically significant coarctation.

Although often considered benign, pseudocoarctation has been occasionally associated with complications such as aneurysm formation, aortic dissection, and compression of adjacent mediastinal structures [5,7]. In this case, transthoracic echocardiography revealed mild dilatation of the proximal ascending aorta with trivial aortic regurgitation. While these findings were not directly attributable to the site of kinking, they underscore the importance of comprehensive cardiovascular evaluation, as concomitant aortic abnormalities may coexist and warrant longitudinal surveillance.

The differential diagnosis between pseudocoarctation and true coarctation remains essential in clinical practice, as misclassification may lead to unnecessary invasive procedures or inappropriate long-term management strategies. The absence of upper-to-lower limb blood pressure gradient, lack of collateral circulation, and absence of clinical signs of aortic obstruction were decisive in establishing the correct diagnosis in our case. The coexistence of pulmonary embolism and aortic kinking in this patient was considered incidental. To date, no causal association between pseudocoarctation of the aorta and venous thromboembolic disease has been reported in the literature [3,4]. The pulmonary embolism was therefore managed independently according to standard therapeutic protocols.

Management of pseudocoarctation is generally conservative in asymptomatic patients without associated complications [3,5,6]. Surgical or endovascular intervention is reserved for selected cases presenting with aneurysmal degeneration, progressive symptoms, or significant associated vascular pathology. Although pseudocoarctation is generally considered a benign condition, long-term clinical and imaging follow-up is recommended because of the potential risk of aneurysm formation and other aortic complications reported in the literature [5-7]. In our patient, conservative management with periodic clinical and imaging follow-up was chosen in accordance with current recommendations.

This report highlights the diagnostic challenges of thoracic aortic kinking, particularly when discovered incidentally during imaging performed for unrelated conditions. Careful integration of clinical, imaging, and echocardiographic findings is essential to avoid misdiagnosis and ensure appropriate management.

## Conclusions

Thoracic aortic kinking is a rare congenital vascular anomaly that may mimic true aortic coarctation on imaging studies. Accurate recognition of its characteristic clinical and radiological features is essential to avoid misdiagnosis and unnecessary investigations or interventions. Although usually benign, long-term follow-up is recommended due to the potential risk of delayed aneurysmal changes. As a single case report, our findings should be interpreted with caution but highlight the importance of recognizing this uncommon entity and its characteristic imaging features in clinical practice.
